# Intralipid therapy and adverse reproductive outcome: is there any evidence?

**DOI:** 10.1530/RAF-20-0052

**Published:** 2021-06-03

**Authors:** Parijot Kumar, Kevin Marron, Conor Harrity

**Affiliations:** 1 Beaumont Hospital, Dublin, Ireland; 2 RCSI University of Medicine and Health Sciences, Dublin, Ireland; 3 ReproMed, Dublin, Ireland

**Keywords:** intravenous intralipid, meta-analysis, assisted reproduction, pregnancy loss, reproductive immunology

## Abstract

Controversy exists regarding the benefits of intravenous intralipid therapy in patients with a poor reproductive history. It is frequently reported that there is no evidence to support the effectiveness, utility or safety for this treatment. While individual studies may be perceived as weak, a systematic review and meta-analysis were performed to determine if there is any advantage to patients. PubMed, Embase and Scopus searches were performed with the target populations being either recurrent pregnancy loss (RPL), or recurrent implantation failure (RIF) undergoing assisted reproductive technology (ART) and receiving intralipid infusions. These cohorts were compared with either placebo, no intervention or alternative treatments. The most relevant outcome measures were considered to be clinical pregnancy rate (CPR), live birth rate (LBR), implantation rate (IR) and miscarriage rate (MR). Twelve studies encompassing 2676 participants met the criteria for selection and were included and reviewed. Treatment of the target population with intralipid led to an improvement in IR (Odds Ratio (OR): 2.97, 2.05–4.29), pregnancy rate (OR: 1.64, 1.31–2.04), and LBR (OR: 2.36, 1.75–3.17), with a reduction in MR (OR: 0.2, 0.14–0.30). Although intravenous intralipid is not recommended as a routine treatment for recurrent miscarriage or implantation failure, there is enough data to suggest consideration in selected patients where routine testing is unremarkable, standard treatments have failed and immunological risk factors are present. The presence of abnormal uterine natural killer (uNK) cells needs more study as a target marker to determine those who could benefit.

**Lay summary:**

There is controversy regarding the benefits and efficacy of intravenous intralipid therapy in patients with a poor reproductive history. It is frequently reported that there is no credible evidence to support their use. A situation we frequently face as medical professionals is patients asking us to consider immune therapy (such as intralipid) for reproductive failure where good quality embryos have been used. Intralipid infusions have been reported to improve pregnancy rates with IVF, and reduce the miscarriage risk in selected patient groups, but study results are not universally accepted. We have performed a detailed review and analysis of the literature to determine if there is any benefit to this immune treatment in specific patient groups. Our paper identified and analyzed 12 studies, finding that treatment with intravenous intralipid leads to an improvement in implantation, pregnancy and live birth rates, with a decrease in miscarriage rate. This study shows that there is evidence to suggest consideration of intralipid in certain patients where standard treatments have failed.

## Introduction

Repeated Implantation failure (RIF) and recurrent pregnancy loss (RPL) are among the most challenging scenarios in reproductive medicine. The cause often remains unknown, leading to frustration and stress. As a result, some patients or physicians seek treatments without a strong supporting evidence base. It is important to fully evaluate these therapies, with a comprehensive discussion regarding potential risks and benefits. Assisted Reproduction has many of these 'Add-Ons', whose value has not been confirmed, often with additional cost implications (Macklon *et al.* 2019). One such intervention is intralipid (IL) to improve pregnancy outcomes. Causes of miscarriage can be difficult to elucidate and are often multifactorial. Chromosomal abnormalities are the main cause of early pregnancy failure, and the probability of a euploid embryo decreases with female age. Anatomical factors, infection, endocrine and thrombophilic abnormalities may also play a role (Ford & Schust 2009, Pirtea *et al.* 2021, Rimmer *et al.* 2021). Implantation failure is a separate entity but with partially overlapping causes and treatments (Christiansen *et al.* 2006). The etiological factors of RIF are again primarily related to the embryo or endometrium, but may also include disorders of the implantation window and endometrial receptivity. Immunological factors remain a controversial cause for both failed implantation and pregnancy loss.

Early in the study of reproductive immunology, the role of peripheral blood natural killer (pNK) cells (CD16+/56+^dim^) in pregnancy failure was suggested (Wegmann *et al.* 1979). Peripheral and uterine natural killer cells (uNK, CD16-/56+^bright^) have very different phenotypes and functions (Koopman *et al.* 2003, Horowitz *et al.* 2013) leading to much confusion and misunderstanding. Over time a shift occurred, focusing on the endometrium and uNK cells. Multiple studies have shown that RIF/RPL are associated with elevated NK cell numbers and activity, or higher concentrations of certain T-lymphocyte subpopulations (Sacks *et al.* 2012, Tang *et al.* 2013, Dakhly *et al.* 2016). Many leukocyte subsets possess the capacity to produce either pro-inflammatory (e.g. TNF-α, IFN-γ) or anti-inflammatory (e.g. IL-10) monomeric cytokines (Jung *et al.* 1993). Stimulated cytokine expression in CD4+ T cells shows an association between increased expression of Th1 markers and the incidence of RPL/RIF (Kwak-Kim *et al.* 2003). Pro-inflammatory cytokines are thought to stimulate NK cell activatory KIR receptors, rendering them more cytotoxic (Szereday *et al.* 1997).

Intralipid, developed in 1961, is a fat emulsion containing soybean oil, glycerin and egg phospholipids, used intravenously as part of parenteral nutrition in patients unable to tolerate an oral diet (Wretlind 1981, Driver *et al.* 1989, Granato *et al.* 2000, Meng *et al.* 2016). The main components are polyunsaturated fatty acids like linoleic, α-linolenic, oleic, palmitic and stearic acid (Ota *et al.* 1985, Shreeve & Sadek 2012, Abdolmohammadi-Vahid *et al.* 2016). A series of serendipitous observations suggested immunosuppressive properties. Increased bacteremia risk was seen in neonates using lipid-based parenteral nutrition (Jarvis *et al.* 1983, Freeman *et al.* 1990, Bansal *et al.* 2012). Intravenous lipid use also increased the risk of infective complications in surgical patients (Snydman *et al.* 1982). Lower rates of graft vs host disease after bone marrow transplantation were found in patients using soybean oil-based parenteral nutrition (Muscaritoli *et al.* 1998). Research in a reproductive setting inevitably followed. A study to assess trophoblast membrane vesicles for treating recurrent miscarriage found a greater success in the control group receiving IL (Johnson *et al.* 1991).

The unique hemochorial human placenta, and lack of a suitably similar animal model, means that RIF/RPL, have not significantly benefited from the insights that can be gained from studies of this nature. An early murine abortive model (CBAxDBA/2) identified a link between TNF-α, IFN-γ, IL-2 and spontaneous miscarriage (Wegmann 1990). Mouse models show a key role for Tregs in the tolerance paradigm (Zenclussen *et al.* 2005), but this is not easily shown in humans. Mating studies also show that IL can be highly effective at preventing abortion in mice, and that this protection is prolonged (Chaouat *et al.* 1990, Clark 1994). Human research demonstrates that IL can modulate immune function by inhibiting NK cell cytotoxicity (Coulam & Acacio 2012, Meng *et al.* 2016, Placais *et al.* 2020) by impairment of the macrophage antigen presentation function (Tezuka *et al.* 1988). A significant fall in NK activity and lymphokine-activated killer activity can be seen after IL administration (Sedman *et al.* 1991), and reduction in pro-inflammatory mediators produced by Th1 cells (Granato *et al.* 2000, Zenclussen *et al.* 2005, Abdolmohammadi-Vahid *et al.* 2016).

If classical causes for RIF/RPL are found, it is not unexpected that immunotherapy will not improve outcomes. It is established that only patients whose reproductive failure has an identifiable immunologic factor would be expected to respond to immunotherapy (Coulam 2020). Proper selection of appropriate patient subgroups within these diverse syndromes remains challenging. This systematic review and meta-analysis aims to report if there is any evidence of improving pregnancy outcomes with IL in women with RPL and/or RIF based on a compilation of existing knowledge. Additional aspects to determine where if immunological parameters exist which could be used as markers to indicate in which cases IL may have a beneficial role, and to assess if their efficacy has been compared with other immunotherapy agents.

## Methods

### Literature search methodology

PubMed, Embase and Scopus were the primary databases for literature search. Combinations of Medical Subject Headings (MeSH) and keywords were used to create subsets, including 'pregnancy' OR 'miscarriage' OR 'assisted reproduction' (ART) OR 'IVF' (in vitro fertilization) OR 'ICSI' (intra cytoplasmic sperm injections) OR 'implantation' OR 'Natural Killer' (NK). The subset was combined with 'Intralipid' using 'AND', to produce citations applicable to the research question. Searches included books, documents, clinical trials, meta-analysis, randomised controlled trials and reviews. No language restrictions were placed. Publications from 1949 to 2020 were included. To confirm all literature was reviewed the references and citations from primary papers and similar review articles were hand searched, along with a conference proceeding search to uncover any gray literature, which yielded a further 36 papers.

### Selection process

Potential studies were selected in a two-step process. Citations identified by searches were scrutinized by title/abstract, then full manuscripts were obtained for those meeting the inclusion criteria. Studies were selected if the target populations were women with RPL or RIF ± IVF/ICSI treatment, using IL infusions. The cohorts were compared with placebo, no intervention or alternative treatment. Outcome measures were clinical pregnancy rate (CPR), live birth rate (LBR), implantation rate (IR) and/or miscarriage rate (MR). A total of 3032 studies were found on preliminary search. Once duplicates were removed 2369 remained for screening and eligibility assessment. The final decision for inclusion or exclusion of articles was done by a thorough manuscript evaluation, with 57 studies for eligibility assessment from which 45 were excluded. Twelve studies (*n* = 2676, 1592 controls and 1084 treated with ILs) met the criteria for selection and were included for review.

### Data extraction

Two authors (P K and C H) independently reviewed the manuscripts to determine if inclusion criteria were met. Data was extracted and compiled as per study parameters. Information collected included authors, publication year, study design, inclusion criteria, patient size, intervention used, controls, alloimmune assessment, and outcome measures. Predesigned forms were created to extract aggregate data. When missing data were encountered, attempts were to obtain individual data from the corresponding author.

### Statistical analysis

Review Manager (RevMan v5.6) software (Cochrane Collaboration, Oxford, UK) was used for meta-analysis. A traditional weighted average meta-analysis was calculated, yielding a Mantel-Haenszel odds ratio with 95% CIs, and heterogeneity investigated with I^2^ statistics. An odds ratio (OR) >1.0 indicates a positive benefit, with CIs, representing the degree of uncertainty, to assess if a difference is significant. Considerable heterogeneity was acknowledged as an I^2^ statistic of 75–100%.

## Results

### Study characteristics

Twelve studies were included for analysis, with six RCTs (El-Khayat & Sadek 2015, Check & Check 2016, Dakhly *et al.* 2016, Meng *et al.* 2016, Singh *et al.* 2019, Al-Zebeidi *et al.* 2020). Remaining papers included five cohort studies (Coulam & Acacio 2012, Harrity *et al.* 2018, Martini *et al.* 2018, Ehrlich *et al.* 2019, Placais *et al.* 2020) and one non-randomized trial (Ndukwe 2011). The non-randomized study was deemed suitable due to well applied inclusion criteria (≥3 failed transfers), a defined abnormality on immunological testing (elevated TH1:2 cytokine ratios), and relevant outcome measures. As with many other systematic reviews, high heterogeneity, and quality variations of the individual studies mean the overall results must be fully assessed and interpreted with care. There were a number of aspects to identify in these studies and their differences were to be noted. Study characteristics and outcome measures are described in Table 1. There were 2676 participants evaluated, and subgroup analyses were performed to identify associations and trends.

**Table 1:** Included studies and characteristics.

Study	Study design	Inclusion criteria	Patients		Type of intervention		Additional medications	Outcome measures	Sig.
			Cont.	Int.	Cont.	Int.			
Check and Check (2016)	Matched control	Age group 40–42 years; history of RPL or RIF	10	10	No treatment	Intralipid (4 mL diluted at 20% in 100 mL saline) infusion over 1 h	None	CPR; LBR; MR	No
Meng *et al.* (2016)	RCT	≥3 unexplained miscarriages before 12th gestational week; peripheral NK cells >20%	78	76	IVIG 25 g infusion over 8 h	Intralipid (20% in 250 mL saline) infusion over 2 h	None	CPR; LBR	No
Placais *et al.* (2020)	Cohort study	≥3 recurrent miscarriages before 12th gestational week and/or ≥3 implantation failures of ≥2 good embryos transfers; absence of any cause of RPL or RIF	36	26	Placebo	Intralipid infusion	Low dose aspirin; prednisolone (10 mg/day); progesterone; vitamin D	CPR; LBR	Yes
Martini *et al.* (2018)	Cohort Study	≥3 unexplained miscarriages or infertility; peripheral NK cells >19%	20	127	Placebo	Intralipid (4 mL diluted at 20% in 250 mL saline) infusion over 90–120 min	None	CPR; LBR	No
Dakhly *et al.* (2016)	RCT	≥3 unexplained miscarriages or infertility; peripheral NK cells >12%	152	144	Saline (250 mL) infusion	Intralipid (2 mL diluted at 20% in 250 mL saline) infusion over 30–60 min	None	CPR; IR; LBR; Chemical pregnancy rate	No
Harrity *et al.* (2018)	Cohort study	History of RIF and/or RPL	134	134	No treatment	Intralipid (20%) infusion	Prednisolone 15–25 mg; Omega 3.3 g; B complex; vitamin D3; LMWH	CPR; IR; MR	Yes
Coulam and Acacio (2012)	Cohort study	History of unexplained infertility, RIF, RPL	242	200	IVIG	Intralipid infusion	None	CPR; LBR	No
Ndukwe (2011)	Non-randomized study	≥3 implantation failures with elevated TH1:TH2 cytokine ratios	46	50	No treatment	Intralipid (20%) infusion	None	CPR	Yes
El-Khayat and Sadek (2015)	RCT	Failure to achieve pregnancy after 2–6 ICSI cycles with the transfer of ≥10 high-grade embryos	102	101	No treatment	Intralipid (20%) infusion	None	CPR; IR; LBR	Yes
Al-Zebeidi *et al.* (2020)	RCT	Age < 42 years with BMI < 30 kg/m^2^; ≥3 RIF undergoing ICSI cycles	71	71	No treatment	Intralipid (100 mL diluted at 20% in 500 mL saline) infusion over 150 min	None	CPR; LBR	Yes
Singh *et al.* (2019)	RCT	Age group 20–40 years; with primary infertility undergoing nondonor oocyte IVF/ICSI with at least one previous implantation failure	50	52	Normal saline	Intralipid (4 mL diluted at 20% in 250 mL saline) infusion	None	Biochemical pregnancy rate; CPR; LBR; take home baby rate	Yes
Ehrlich *et al.* (2019)	Cohort study	History of repeated unsuccessful IVF cycles and pre-viable pregnancy loss	651	93	No treatment	Intralipid (100 mL diluted at 20% in 500 mL saline) infusion over 3–4 h	Prednisolone; LMWH; aspirin; heparin	CPR; LBR	No

Cont, control; CPR, clinical pregnancy rate; Int, intervention; IR, implantation rate; IR, implantation rate; MR, miscarriage rate; Sig, significant difference.

### Intralipids vs no intervention (control/placebo)

**Figure 1:**
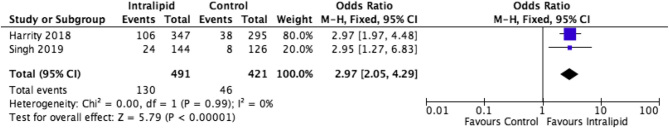
Effect of Intralipid on IR vs control, all studies, all patients.

**Figure 2:**
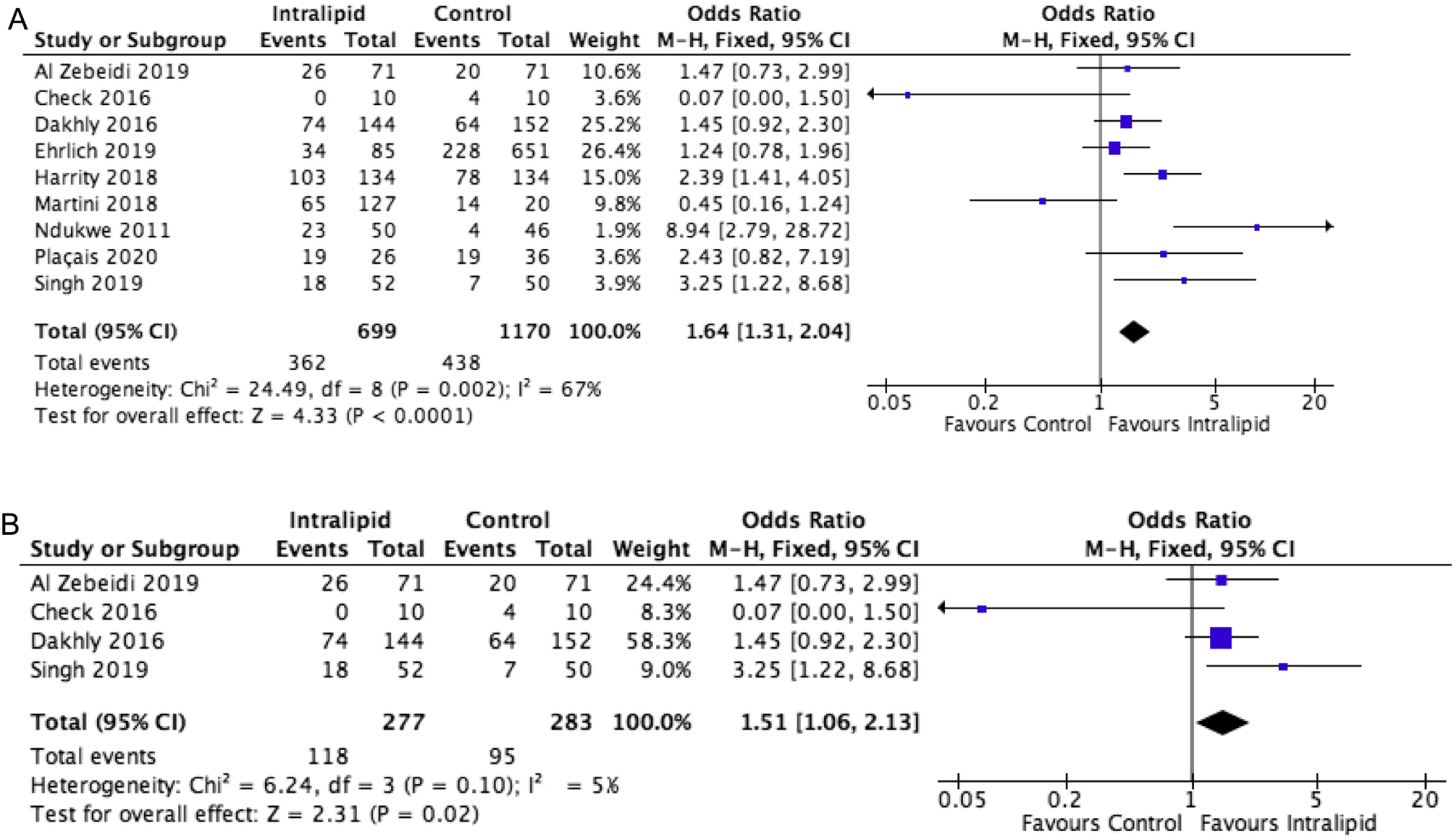
(A) Effect of intralipid on CPR vs control, all studies, all patients. (B) Effect of intralipid on CPR vs control, all studies, RCTs.

**Figure 3:**
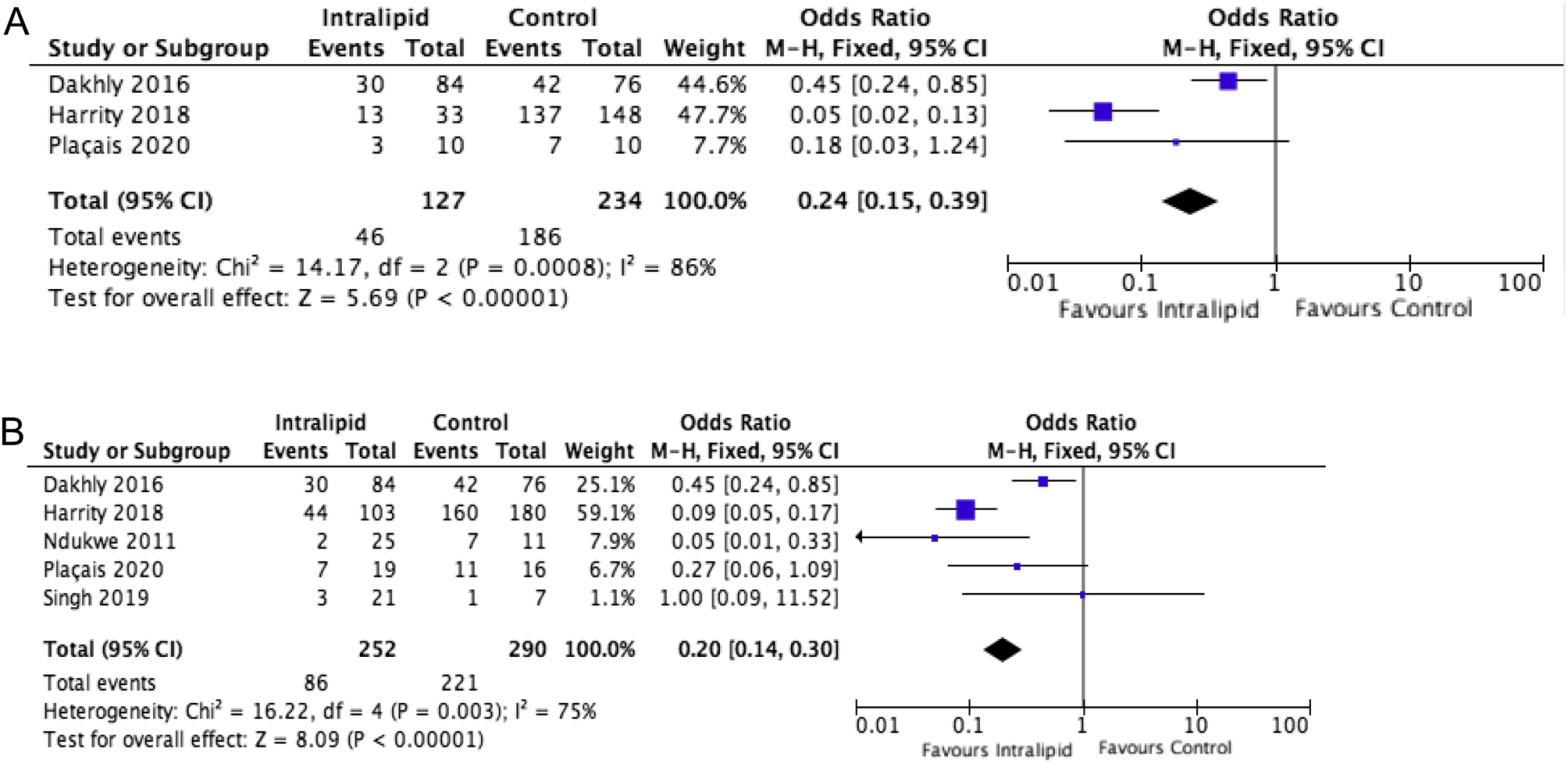
(A) Effect of intralipid on MR vs control, RPL only. (B) Effect of intralipid on MR vs control, all studies.

**Figure 4:**
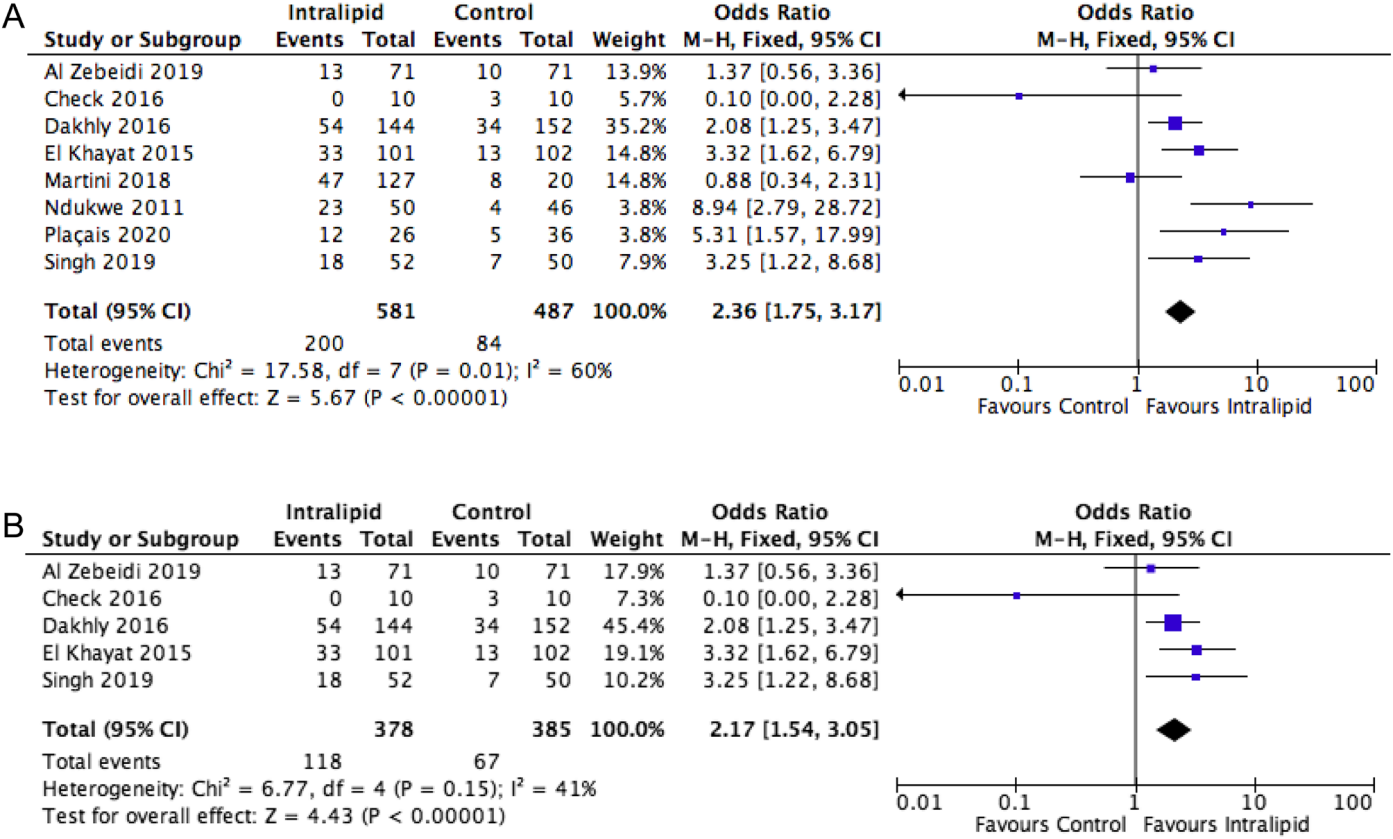
(A) Effect of intralipid on LBR vs control, all studies, all patients. (B) Effect of intralipid on LBR vs control, all studies, RCTs.

Analysing data from all studies and all patient subgroups (RPL and RIF) comparing IL vs placebo or no treatment identified ten trials, including five RCTs, with 2072 cases. Key outcome measures to compare were IR, CPR, MR and LBR. The beneficial, and statistically significant, effects of IL on these outcomes are demonstrated (Figs 1, 2, 3 and 4).

#### Implantation rate

IR, or the number of gestational sacs seen on ultrasound divided by the number of embryos transferred, is an important performance indicator for ART centers. A benefit is compensation for differences in practice between units using elective single embryo transfer strategies, and those using of multiple embryos. This measure is often not reported, and only two studies provided IR as an outcome following embryo transfer with a regime incorporating IL compared to placebo (*n* =912). Study data reveal a strong and significant improvement in implantation following the addition of IL (OR: 2.97, 2.05–4.29, I^2^ : 0%) (Fig. 1 and Table 2).

**Table 2:** Summary table showing intralipid outcomes across all the included studies, stratified by total trials, randomized control trials only, vs reproductive pregnancy loss only and vs IVIG, the odds ratio (OR) generated in each meta-analysis and the 95% CI generated.

Outcome	Studies	Participants	OR (95% CI)
Vs control (all studies included)			
Clinical pregnancy	*9*	1869	1.64 (1.31, 2.04)
Live birth	8	1068	2.36 (1.75, 3.17)
Miscarriage	5	542	0.20 (0.14, 0.30)
Implantation rate	*2*	912	2.97 (2.05, 4.29)
Vs control (all patients, RCTs only)			
Clinical pregnancy	4	402	1.83 (1.19, 2.80)
Live birth	5	763	2.17 (1.54, 3.05)
Vs control (RPL cases only, all studies included)			
Clinical pregnancy	3	428	1.17 (0.78, 1.74)
Live birth	3	529	2.67 (1.79, 3.98)
Miscarriage	3	361	0.24 (0.14, 0.30)
Vs IVIG (all studies included)			
Live birth	2	634	1.02 (0.74, 1.40)

#### Pregnancy rate

##### All studies

Systematic review identified nine studies (*n* = 1869) reporting clinical pregnancy rate as an outcome measure. A significant improvement in CPR (OR: 1.64, 1.31–2.04, I^2^ : 67%) is found with IL use compared to no immunotherapy in the patient populations (Fig. 2A).

##### Randomized trials

When cohort studies are excluded, four randomized trials remained (*n* = 402). Outcomes from RCTs alone remain similar, and support intervention, with a statistically significant improvement in CPR (OR: 1.51, 1.06–2.13, I^2^ : 52%) following IL use, and less heterogeneity in the included studies (Fig. 2B).

#### Miscarriage rate

A subgroup analysis of patients with only RPL was performed, by excluding those diagnosed with RIF or other etiologies, in order to assess if the attempted immunomodulatory regime had any effect on the miscarriage rate (*n* = 361). Interestingly, in this specific group of RPL cases, the published data clearly demonstrate that the addition of IL into the treatment has no impact on the ability to achieve a clinical pregnancy, with no difference in CPR between IL and control groups (OR: 1.17, 0.78–1.74, I^2^ : 82%). This would not be unexpected, as these patients, by definition, do not have an issue with infertility/implantation at presentation. In this population, the primary concern is if IL therapy could reduce the risk of first-trimester loss, improving the probability of subsequent delivery. Meta-analysis of study data demonstrate a very significant reduction in MR (OR: 0.24, 0.15–0.39, I^2^ : 86%) (Fig. 3A) and improvement in subsequent LBR (OR: 2.67, 1.79–3.98, I^2^ : 53%) with immunotherapy. The use of IL treatment does appear to be beneficial at reducing pregnancy loss in this high-risk population. The overall miscarriage rate for all patients, without selection by etiology, was also assessed (*n* = 542). Again, study data demonstrates a significant reduction in MR with intralipid use (OR 0.20, 0.14–0.30, I^2^ 75%) (Fig. 3B).

#### Live birth rate

##### All studies

Live birth rate (LBR) is the key measure by which many interventions in assisted reproduction are judged. Eight studies (*n* = 1068) reported this important outcome. Interestingly, there is a clinically relevant, and statistically significant, improvement in LBR (OR: 2.36, 1.75–3.17, I^2 :^60%) identified following IL therapy, compared to no intervention, in these high-risk patients (Fig. 4A).

##### Randomized trials

An additional analysis was also performed using data from randomized controlled trials only. With the exclusion of the cohort studies, five RCTs (*n* =763) remain. Patient outcomes with randomized trials alone again demonstrate a statistically significant improvement in LBR (OR: 2.17, 1.54–3.05, I^2^ : 41%) with IL use, and a reduction in study heterogeneity (Fig. 4B).

### Intralipid compared to alternative immunotherapy treatments

Although the first study suggesting a role of IL therapy in the treatment of pregnancy loss was an interventional study, there is a paucity of research in this area since. Steroids and intravenous immunoglobulin (IVIG) comprised the early reproductive immunomodulatory regimes, but a transition from IVIG to IL for cost and safety reasons has been seen. A systematic review was performed for studies comparing IL with other immunotherapies.

#### Intralipid vs IVIG

**Figure 5:**
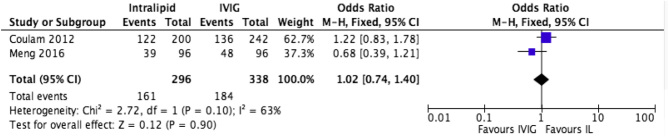
Effect of intralipid on CPR vs IVIG, all studies, all patients.

Suppression of NK cell activity with IVIG has been reported *in vitro* (Ruiz *et al.* 1996, Roussev *et al.* 2007) and *in vivo* (Kwak *et al.* 1996), but whether this translates to improved outcomes is subject to debate. IVIG infusions may enhance LBR by decreased NK killing activity, increased suppressor T-cell activity, suppression of B cell autoantibody production, and actions on Fc receptors (Sewell & Jolles 2002). Several meta-analyses indicate IVIG increases live birth in cases with RPL and positive immunological risk factors (Clark *et al.* 2006, Coulam & Acacio 2012, Li *et al.* 2013, Meng *et al.* 2016). Subgroup analysis suggests improvement in LBR for secondary recurrent miscarriage, but not in primary RPL (Hutton *et al.* 2007). IVIG is not without risk, side effects range from headaches, nausea and vomiting, to more severe allergic reactions (Raziel *et al.* 1996, Meng *et al.* 2016). Immunoglobulins are produced from multiple blood donors, which is a rare risk of infection such as Hepatitis B, C, or HIV (Raziel *et al.* 1996). Due to high costs, the debate over efficacy, and potential adverse effects, IVIG has never been a recommended treatment. IL has gained interest as a safer, more acceptable choice and is shown to directly suppress NK cytotoxicity with equal efficacy as IVIG by *in vitro* (Ruiz *et al.* 1996) and *in vivo* (Roussev *et al.* 2008) assays. Literature review identified two studies (*n* =634) comparing IVIG against IL, with LBR as the primary outcome. No difference in LBR was identified (OR: 1.02, 0.74–1.40, I^2^ : 63%), suggesting equal efficacy (Fig. 5 and Table 2), suggesting IL to be at least as successful as IVIG in treating immune-mediated pregnancy loss in screened patients.

#### Intralipid vs corticosteroids

Clinical benefits of oral corticosteroids in patients with adverse reproductive outcomes have been reported, but despite this, there is no consensus of therapeutic benefit. Steroid use is not recommended by most professional societies. Unfortunately, no trials were identified to compare prednisolone or other glucocorticoids with IL. A retrospective study using prednisolone to treat overactive endometrial immune profiles was identified but reported a beneficial effect on parameters in only 54.5% of cases, with a paradoxical deleterious effect in 29% where the immune profile actually worsened (Ledee *et al.* 2018*a*). Comparative interventional studies are needed to contrast the impact of steroids and IL.

### Intralipid use and abnormal immunological assessment

A major criticism with reproductive immunotherapy has been patient selection criteria, or lack of. Ideally there should be a combination of treatment failure with a euploid embryo and proven abnormal markers on auto/alloimmune assessment before considering immunomodulation. A review to determine if any specific immunological abnormalities could predict a better response to IL treatment (Table 3) and analyze the effect of IL on individual immunological parameters (Table 4) is described.

**Table 3:** Summary table showing intralipid outcomes vs no intervention across all the included studies, stratified by the use of immunological testing as an inclusion criteria.

Diagnostic test	Threshold level	CPR	*P*	LBR	*P*
uNK (endometrium)					
No comparative studies	NA	NA	–	NA	*–*
pNK (blood): two studies					
1 >12%	>19%	0.51 vs 0.70	0.12	0.37 vs 0.40	0.80
1>19%	>12%	0.58 vs 0.50	0.15	0.38 vs 0.22	0.005
Th1:2 cytokine ratio (blood): two studies					
2 CPR and 1 LBR	Elevated^1^	0.66 vs 0.46	<0.001	0.46 vs 0.10	<0.001

^1^Elevated TH1:2 cytokine ratio defined as TNFa:IL-10 > 30.6 and/or IFNg:IL-10 > 20.5. NA, not assessed.

**Table 4:** Summary table showing a change in immunological testing parameters before and after intralipid treatment using data from Meng 2016 (*n* =79) and Ledee 2018 (patients achieving pregnancy, *n*  =27).

Diagnostic test	Pre-IL	Post-IL	*P*
pNK conc (% CD56+CD16+)	26.1	24.4	<0.001
pNK cytotoxicity (K562 assay)	37.8	26.6	<0.001
uNK count (CD56 cells/field)	62.8	45.5	0.04
uNK activity (IL-15:Fn-14)	3.6	0.79	<0.001
Uterine TH1:2 (IL-18:TWEAK)	0.18	0.08	<0.001

#### Uterine natural killer cells

Although uNK play an important role in the maternal immune response to pathogens, they are primarily involved in trophoblastic invasion and angiogenesis (Khan *et al.* 2019). There is conflicting data on the impact of uNK cell concentrations and fertility, suggesting function, not just numbers, may be associated with pregnancy loss (Coulam 2020). Endometrial biopsy for uNK assessment is proposed as a marker for reproductive failure, but the literature review did not identify any studies testing this hypothesis in terms of patient selection for IL in combination with controls. An observational study was identified assessing uNK numbers (CD56 cells) by biopsy, uNK maturation and activity (IL-14/FN-14 ratio) and the local Th1:2 Balance (Il-18:TWEAK) (Ledee *et al.* 2018*a*). In this trial a high LBR of 54% (51/94) was achieved in poorer prognosis RIF patients with overactive endometrial profiles following IL treatment. There was also a significant decrease in each of the three biomarkers used to diagnose over-immune endometrial activation after IL use (Ledee *et al.* 2018*a*). The lack of a control/placebo group limits the interpretation of the results.

#### Peripheral blood natural killer cells

An association between pNK concentration/activity and the adverse reproductive outcome is proposed but a shortage of data using these markers as inclusion criteria for interventional trials is seen. Meta-analysis demonstrates higher pNK numbers in RPL cases compared to controls, but no difference in IVF LBR with elevated pNK numbers/activity (Seshadri & Sunkara 2014). CD56+ve pNK cells typically comprise around 10% of peripheral blood lymphocytes (Robertson & Ritz 1990), but reliable reproductive reference ranges have not been established, and no agreement on an upper threshold; pNK ≥12% has been associated with poor reproductive outcome (Michou *et al.* 2003, Thum *et al.* 2004), but alternatively pNK >18% are reported as specific for RPL/RIF (King *et al.* 2010). Two studies used elevated pNK levels as inclusion criteria. Using a cut-off of 19%, no benefit was seen in CPR or LBR with IL treatment. Strangely, when a lower threshold of 12% was used, there was a significant improvement in LBR, although CPRs were no different. There is insufficient evidence to establish any conclusion regarding the use of pNK levels as a selection tool for IL use.

#### Th1:Th2 cytokine ratios

Th1:Th2 ratios have been associated with reproductive failure; Th1 cytokine excess may be detrimental for implantation and placental development, with similar issues suspected in the absence of Th2 cytokines (Chaouat 2007). Measurement of stimulated intracellular cytokine expression in CD4+ T cells has been suggested to assess this. Systematic review identified two studies using elevated Th1:Th2 cytokine ratios for patient selection. A non-randomized trial reported a significant improvement in CPR and LBR with IL treatment in cases with elevated cytokines (Ndukwe 2011), and a cohort study reported improvement in IR, CPR and reduction in MR. No studies were identified that reported changes in pre- and postinfusion Th1:Th2 cytokine ratios to measure the *in vivo* effect of IL treatment.

## Discussion

Although it is frequently reported that there is no evidence to support IL use for the adverse reproductive outcome, literature review demonstrates a growing body of published data, with 12 studies (including six RCTs) designed to answer important and relevant questions. Meta-analysis reveals higher IR, CPR and LBR with IL, and a reduction in MR, with all differences statistically significant. For certain obstetric complications, such as pregnancy-induced hypertension, pre-eclampsia, or intrauterine growth restriction, immunological maladaptation plays a significant role (Savasi *et al.* 2016). Although there is a clear understanding that normal endometrial immunological function, in particular uNK cells, is needed to allow implantation and early pregnancy development (van Mourik *et al.* 2009), there is much to learn about the consequences of changes in leukocyte numbers or activity. As a result, there remains great debate as to which tests or treatments, if any, should be recommended.

Attempts to study peripheral blood lymphocytes as a marker of uterine immune dysfunction are controversial. Although pNK cells are reported to be elevated in women with RPL (Yamada *et al.* 2003), not all studies agree (Emmer *et al.* 1999, Souza *et al.* 2002, Wang *et al.* 2008). A criticism of hematological testing is that no physiological reason for a relationship between blood and endometrial NK levels should exist (Moffett-King 2002), and analysis shows no correlation between pNK and uNK concentrations (Clark 2010). Blood values are influenced by external dynamics and prone to fluctuations, so they tend to lack scientific credibility (Maecker *et al.* 2012, Moffett & Shreeve 2016). Moves to focus on the uterine environment are increasing. Early endometrial analysis identified RPL cases have different patterns of CD4 and CD8 cells, an increase in percentage CD56^dim^ NK cells, and more B Lymphocytes (Lachapelle *et al.* 1996). RPL patients have more uNKs than controls, and prednisolone can significantly reduce the number of endometrial CD56 cells (Quenby *et al.* 2005). Differential gene expression patterns show that endometrial immune profiles are dysregulated in RIF/RPL (Ledee *et al.* 2016). Changes in mRNA cytokine ratios, specifically IL-15/Fn-14 as a biomarker of uNK cell activation and ILI8/TWEAK to assess Th1:Th2 balance, can identify immune over/under activation, with an imbalance in these ratios in over 80% of RIF cases (Ledee *et al.* 2016). Endometrial flow cytometry demonstrates higher uNK levels in RIF, while B, pNK, and NK-T cells are higher in RPL (Harrity *et al.* 2019, Marron *et al.* 2019). An endometrial decidualization profile has been proposed, incorporating molecular analysis of decidualization/implantation factors (FOXO1, GZMB, IL15, SCNN1A, SGK1, SLC2A1) to calculate a score identifying patients that may benefit from intervention (Wolff *et al.* 2003, Salker *et al.* 2011, Ruan *et al.* 2012, Vasquez *et al.* 2018, Coulam *et al.* 2020). NK activity can be significantly decreased by IL (Roussev *et al.* 2007). The effects can be lasting, with a duration of 4-9 weeks (Roussev *et al.* 2008). Other studies demonstrated decreased *in vitro* activation of T cells, and a reduction in cytokine secretion, with decreased TNF-α, IL-2 and IL-1β (Granato *et al.* 2000, Ledee *et al.* 2018*b*). IL shows promise as treatment for overactive endometrial immune profiles (Ledee *et al.* 2016, Ledee *et al.* 2018*b*). Conversely, stimulatory measures should be employed and immunosuppression avoided in underactive profiles.

Reproductive immunotherapy should only be utilized if there is an identifiable alloimmune cause. If aneuploidy, systemic disease, endometrial receptivity or anatomical factors are the primary issue, then it is not surprising that immunomodulation is of no benefit. Inappropriate use, often at significant additional cost to patients, has done much reputational damage to this field. Older studies that attracted criticism often did not employ screening tests to identify patients with relevant immunological factors. Increasing the use of preimplantation genetic screening (PGT-A) can improve the LBR per transfer by excluding aneuploid embryos. Interestingly, PGT-A has shown that transfer of chromosomally normal blastocysts is not always successful, with at least 30% of euploid embryos failing to implant or ending with miscarriage (Franasiak & Scott 2017). This may identify candidates for personalized screening and consideration of immunomodulation.

Although there is skepticism, only a single study demonstrates a disadvantageous effect of IL on outcome. Inclusion criteria was female age 40–42 years, and HFEA data reports a LBR of only 11.3% in these patients (Human Fertilization and Embryology Authority 2020). A higher LBR of 30% was seen in the controls, compared to 0% receiving IL. Aneuploidy is the most likely cause for implantation failure, and PGT-A was not incorporated into the cycles, with around 58–75% of embryos aneuploid at this age (Franasiak *et al.* 2014). Screening for alloimmune risk factors was also not used in the selection process. The authors hypothesized that IL may indeed be detrimental in older age groups, and this question would warrant more investigation.

Cost is a major benefit of IL compared to other immunomodulation agents. The product cost for Intralipid 20% is around €7.45 per 100 mL infusion (MIMS 2020) in Ireland. Total treatment cost is considerably higher, incorporating other factors such as staffing, health care professional time, expendables (eg cannulas), and the bed cost to administer the infusion. In the UK prices quoted range from £200–300 by IVF clinics. In comparison, IVIG is significantly more expensive, with costs of $7000–14,000 per infusion in the US (Martini *et al.* 2018), or £1700–£2000 per 25 g in UK fertility clinics. The other major benefit of IL over alternatives is reduced patient risk and less adverse side effects (Martini *et al.* 2018), making it a safer and more acceptable choice. Pregnancy outcomes following IL use show a very low rate of adverse events, indicating that it is a safe agent to administer in a RIF/RPL population (Ehrlich *et al.* 2019). Other studies also report no adverse maternal outcomes (Meng *et al.* 2016), further supporting the safety profile.

There are still questions, but emerging data shows that IL treatment can improve outcomes in certain groups. Uncertainty persists in that we do not know the optimal diagnostic tests or threshold levels to clearly identify candidates. Like many unlicensed interventions in reproductive medicine, the large placebo-controlled trials that are needed for conclusive proof are unfortunately unlikely to arise. This is due to many ART centers existing as private stand-alone institutions without the capacity or willingness to develop such trials in their limited patient populations. There are also prohibitive costs associated with industry lead trials and the subsequent approval/registration process. As a consequence, smaller underpowered or non-randomized studies do less to advance the knowledge base. High levels of study heterogeneity remain an issue, with the majority of I^2^ statistics in the 50–90% range, which may represent substantial heterogeneity. A definitive multicenter study, encompassing a large sample population, with selection criteria based on adverse outcome despite normal cytogenetics, combined with abnormal immune assessment, is the only way to achieve a consensus answer. More also needs to be done to establish the optimal dosage regime and timing of the infusion in relation to implantation, with the number and frequency of repeat infusions still not clearly understood. Perhaps this can be aided by further mechanistic studies to assess which IL regimes can best normalize a diagnosed endometrial immune dysfunction prior to embryo transfer, followed by outcome studies to analyze the effects.

## Conclusion

The meta-analysis identifies that a significant body of evidence exists showing that intralipid infusion can help implantation in those with otherwise unexplained infertility and may improve the LBR in those with recurrent miscarriage in the presence of known immunological risk factors. Although these findings are not enough to establish intralipid use as a routine intervention for RIF/RPL yet, there is a role for strong consideration in selected cases, especially when standard treatment has failed, and identifiable risk factors are present.

## Declaration of interest

The authors declare that there is no conflict of interest that could be perceived as prejudicing the impartiality of the research reported.

## Funding

This research received no specific grant from any funding agency in the public, commercial or not-for-profit sectors.

## Author contribution statement

**Figure 6:**
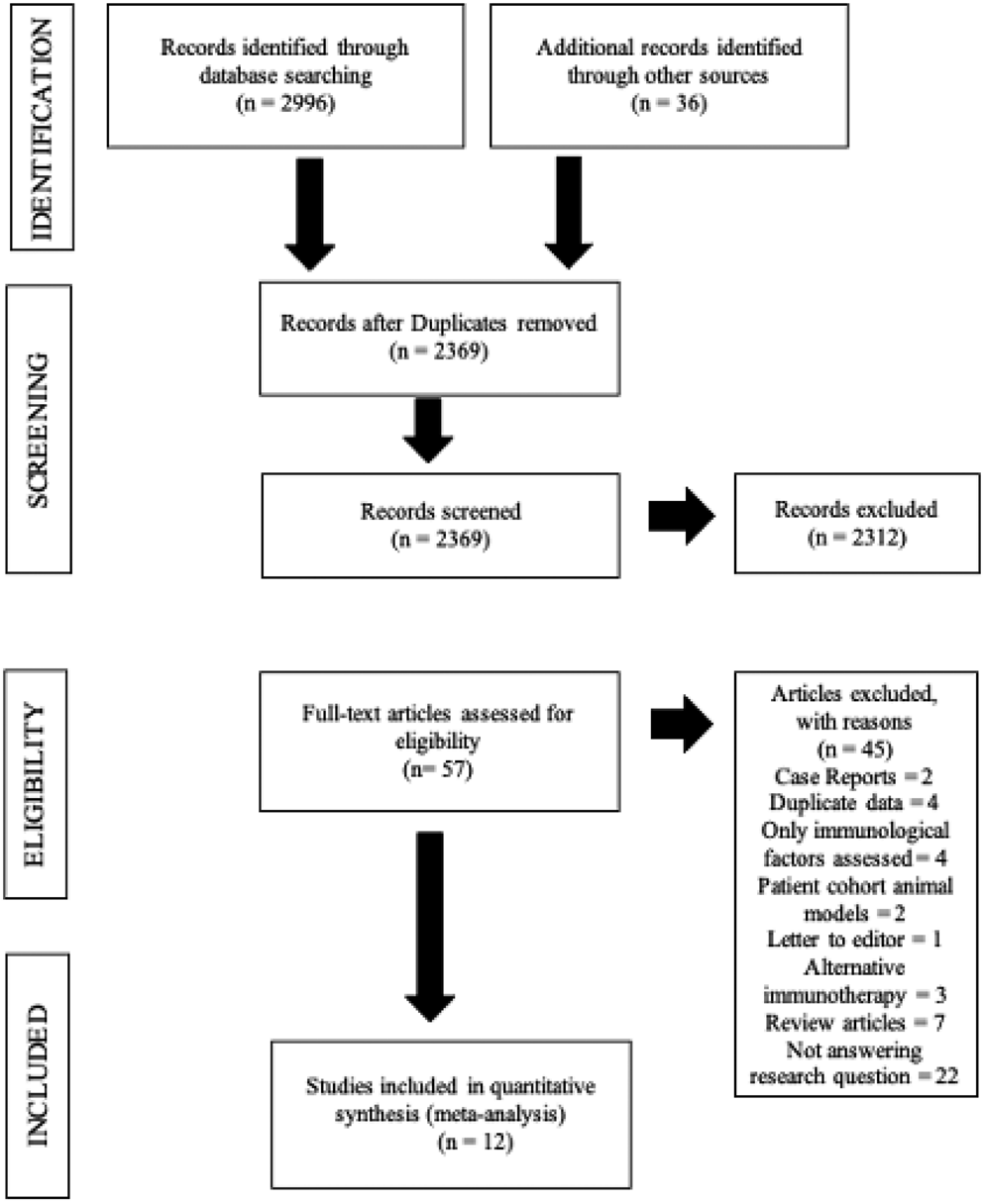
Preferred Reporting Items of Systematic Reviews and Meta-Analysis (PRISMA) flow chart.

Selection and screening process by authors P K and C H. K M contributed to manuscript editing. Process of the literature selection is summarized in PRISMA (Preferred Reporting Items for Systematic Reviews and Meta-Analysis) flow chart (Fig. 6). Any disagreements were resolved by consensus and/or discussion with the senior author (C H).
